# *Cinnamomum cassia* Modulates Key Players of Gut–Liver Axis in Murine Lupus

**DOI:** 10.3390/biomedicines14010006

**Published:** 2025-12-19

**Authors:** Georges Maalouly, Tarek Itani, Nassim Fares

**Affiliations:** 1 Laboratory of Research in Physiology and Pathophysiology, Faculty of Medicine, Saint Joseph University of Beirut, Beirut 1104 2020, Lebanon; georges.maalouly@usj.edu.lb; 2 Faculty of Pharmacy, Saint Joseph University of Beirut, Beirut 1104 2020, Lebanon; tarek.itani@usj.edu.lb

**Keywords:** lupus, microbiota, cinnamon, liver, oxidative

## Abstract

**Background**: Systemic lupus erythematosus is a multi-faceted auto-immune disease. Growing evidence points to gut permeability and microbiota as key players in the development of the disease. *Cinnamomum cassia* is gaining attention as a potential modifier of the gut and liver health. We aim in this study to explore the effect of cinnamon on key elements of the gut–liver axis in imiquimod-induced lupus. **Methods:** Female C57BL/6J mice were stratified into five experimental groups: sham, sham plus cinnamon, lupus, lupus with cinnamon treatment, and lupus with pre- and post-induction cinnamon treatment. Lupus was induced through application of 1.25 mg of 5% imiquimod cream to the right ear, three times per week over six weeks. *Cinnamomum cassia* was given orally at 200 mg/kg, five days weekly. High-Throughput Sequencing of Bacterial 16S rRNA Gene was used on fecal samples with subsequent bioinformatic analysis of microbiota. Western blot and antibody array were used to measure *E. coli* translocation, and hepatic inflammatory, oxidative, and apoptotic markers. **Results**: Cinnamon treatment mitigated the trend toward a lower Firmicutes/Bacteroidota ratio in the lupus mice. While not statistically significant, cinnamon also led to a decrease in Lachnospiraceae abundance. Interestingly, sham mice given cinnamon had more *Lactobacillus* and *Limosilactobacillus*. Furthermore, cinnamon effectively reversed the increase in *E. coli* protein in the liver and normalized the enhanced expression of TLR-7, p-NFκB/NFκB, SOD1 and SOD2 induced by lupus. **Conclusions**: *Cinnamomum cassia* modulates oxidative, inflammatory, and microbial elements of the gut–liver axis in lupus, offering a new perspective on lupus pathogenesis and potential nutritional interventions.

## 1. Introduction

Systemic lupus erythematosus is the prototype of auto-immune diseases, with multiple organ damage. Its pathogenesis is complex and integrates many immunologic, metabolic, and oxidative pathways [1]. Growing evidence points to gut permeability and microbiota as key players in the initiation and development of the disease, with gut–liver axis dynamics characterized by bacterial translocation and activation of TLR4-NFκB in the liver [2].

The human gut microbiota is a complex ecosystem with more than one hundred trillion different microbial organisms [3]. Within this diverse microbial community, more than one thousand bacterial species inhabit the human gut, belonging mostly to four phyla—Firmicutes, Bacteroidota, Proteobacteria, and Actinobacterita. Bacteroidota and Firmicutes dominate the gut microbiota in healthy adults, maintaining a symbiotic relationship with the human host [4]. This ecosystem connects environmental factors with the immune effectors and modulates the immunologic response. When dysbiosis with gut permeability dysfunction occurs, inflammation and immune system sensitization may follow, leading to chronic inflammation and auto-immune diseases [5]. Dietary modification, antibiotics, probiotics supplementation, and fecal microbiota transplantation are emerging as new paradigms to treat or prevent auto-immune or chronic inflammatory diseases.

*Cinnamomum cassia*, a worldwide-used spice, rich in bioactive phytochemicals, is gaining attention as a potential nutritional modifier of the gut microbial structure and gut barrier function [6]. Beneficial effects of cinnamon in lupus have been suggested by recent studies [7] but have never been linked to modulating dysbiosis and liver inflammation in experimental lupus models.

Therefore, this study aims to assess the cinnamon supplementation effect on the gut microbiome, activation of hepatic inflammatory pathways, and liver oxidative stress in imiquimod-induced lupus in mice. Unlike spontaneous genetic models, the imiquimod model relies on the activation of Toll-like Receptor 7 (TLR7) via topical application. This model was selected because it mimics the environmental trigger aspect of human lupus and allows for the study of the innate immune system’s role in initiating systemic autoimmunity [8]. Furthermore, imiquimod-induced lupus presents with well-defined intestinal permeability issues, making it ideal for studying the gut–liver axis [2]. *Cinnamomum cassia* was administered orally at 200 mg/kg. This dose was selected based on previous toxicological assessments confirming its safety in rodents [9,10] and our prior studies demonstrating its efficacy in modulating metabolic and inflammatory pathways without inducing hepatotoxicity [11].

## 2. Materials and Methods

### 2.1. Ethical Statement

The study was conducted with the highest ethical standards for animal care. It was approved by the Ethical Committee of Saint Joseph University (CEHDF 1762, 2 December 2020). The research followed established animal care protocols, including: the Guiding Principles in the Care and Use of Animals by the American Physiological Society, the NIH’s “Guide for the Care and Use of Laboratory Animals” (NIH Publication no. 85–23, revised 1996), and the European Parliament Directive 2010/63 EU, which ensures ethical and humane treatment of animals.

### 2.2. Animals and Study Groups

Fifty adult female C57BL/6J mice were used in this study. Animals were kept in an environment with a constant temperature of 25 °C and humidity of 50 ± 5%, and they experienced a 12:12 h light–dark cycle. They had access to standard rodent food and tap water ad libitum. The experimental design employed stratified block randomization to ensure group equivalence based on baseline body weight. All mice were individually weighed prior to group assignment and ranked from heaviest to lightest. The ranked list was then divided into blocks equal in size to the number of treatment groups, thus creating blocks that were balanced for weight distribution. Within each block, animals were randomly allocated to a single treatment group.

Lupus was induced by skin application on the right ear with 1.25 mg of 5% imiquimod cream three times per week for six weeks. To minimize the stress associated with daily oral gavage—which can independently alter gut microbiota and immune responses—cinnamon was administered via voluntary ingestion [12,13]. *Cinnamomum cassia* powder (Solgar, Leonia, NJ, USA, powder of cinnamon bark and bark extract) was orally administered at 200 mg/kg/day to each animal individually on a small piece of chow slightly moistened with water to retain the cinnamon powder. Furthermore, the experimenter verified each time that the mouse had completely ingested this piece of chow. Accordingly, the animals were divided into five groups of ten mice per group: Sham, Sham treated with cinnamon (Sham Cinna), lupus, lupus treated with cinnamon for six weeks (Lupus Cinna or LC), and lupus treated with cinnamon starting two weeks before and continuously after induction (Cinna Lupus Cinna or CLC, total cinnamon exposure for eight weeks). At the end of the protocol, the animals were anesthetized with a combination of ketamine (75 mg/kg; Interchemie, Waalre, The Netherlands) and xylazine (10 mg/kg; RotexMedica, Trittau, Germany). Once they showed no response to toe pinching, indicating deep anesthesia, they were euthanized for subsequent tissue collection.

### 2.3. Extraction and Purification of Total DNA from Feces

Fecal samples were collected (100 mg to 150 mg) from rectal content to investigate changes in GM composition at the species level. It was notable that all mice, irrespective of the study group, were sacrificed at the same time of day. DNA extraction was performed using the Zymo Quick-DNA™ Fecal/Soil Microbe Miniprep (Zymo Research, Irvine, CA, USA) according to the manufacturer’s instructions. The bead-beating step was achieved using a homogenizer (Biospec, Bartlesville, OK, USA) twice for 2 min. The concentration and quality of DNA was assessed by Nanodrop D100 Spectrophotometer (Nanodrop Technology, Wilmington, DE, USA) and measured using a Qubit dsDNA BR Assay Kit with a Qubit 4.0 Fluorometer (Thermo Fisher Scientific, Inc., Waltham, MA, USA), following the manufacturer’s instructions.

### 2.4. High-Throughput Sequencing of Bacterial 16S rRNA Gene

The microbial community was assessed via the high-throughput sequencing of the V3-V4 region of the bacterial 16S rRNA gene. We used for library preparation the Zymo Research Quick-16S™ NGS Library Prep Kit (Zymo Research, Irvine, CA, USA) according to the manufacturer’s protocol. The resulting PCR products were purified and loaded onto Illumina MiSeq Reagent Kit v3 (San Diego, CA, USA) and sequenced on an Illumina MiSeq instrument with 2 × 300 paired end according to the manufacturer’s instructions. The quality of the run was checked internally using PhiX, and then each pair-end sequence was assigned to its samples with the help of the previously integrated index. Each pair-end sequence was assembled using Flash software v1.2.6 (Magoc 2011) using at least a 10 bp overlap between the forward and reverse sequences, allowing 10% mismatch. The lack of contamination was checked with a negative control during the PCR, using water as a template. The quality of the stitching procedure was controlled using the Zymobiomics Microbial community DNA Standard with 8 bacterial species that were run in parallel to the current samples.

### 2.5. Bioinformatics Analysis

Sequences were analyzed and normalized with the pipeline FROGS (Find Rapidly Operational Taxonomic Units (OTUs) with Galaxy Solution) [14]. PCR primers were removed, and sequences with sequencing errors in the primers were excluded. Reads were clustered into OTUs using the Swarm clustering method. Chimeras were removed, and 988 OTUs were assigned at different taxonomic levels (from phylum to species) using the RDP classifier and NCBI Blast+ on the 16S SILVA 138.1 database.

A total of 81,439 reads were randomly selected for each sample to normalize the data. The sequences were aligned using Clustal Omega 1.1.0 via the profile alignment option in Sea View 4.5 [15]. Neighbor joining trees, as well as maximum-likelihood trees using PhyML 3.1, were built to assess identifications [16].

### 2.6. Western Blot and Antibody Array

Liver tissue was homogenized and lysed in an assay lysis buffer composed of 150 mM NaCl, 50 mM Tris-OH (pH 7.5), 95 mM EDTA, and 0.5% Triton X-100, supplemented with protease and phosphatase inhibitors to extract total protein content. Protein concentrations were determined using the Bradford protein assay (Bio-Rad, Marnes-la-Coquette, France). Samples were then mixed with Laemmli loading buffer (Bio-Rad) containing 10% β-mercaptoethanol (Sigma-Aldrich, St. Louis, MO and Burlington, MA, USA) and incubated at 37 °C for 20 min for denaturation. Proteins were separated on 12% SDS-PAGE gels and transferred to polyvinylidene fluoride (PVDF) membranes (Bio-Rad). Membranes were blocked in TBS–Tween buffer containing 5% BSA, followed by overnight incubation at 4 °C with the appropriate primary antibodies: TLR4 (1/1000; ab22048; Abcam, Cambridge, UK), TLR7 (1/1000; ab24184; Abcam, Cambridge, UK), NFkB (1/1000; 8242; Abcam, Cambridge, UK), and p-NFkB (1/1000; 3033; Abcam, Cambridge, UK).

After being washed with TBS–Tween, the membranes were incubated for one hour at room temperature with goat anti-rabbit and anti-mouse antibodies (1/3000, Bio-Rad Laboratories) and rabbit anti-goat (1/5000. ab6741; Abcam, Cambridge, UK) as secondary antibodies.

To perform the antibody array, 5 µg of protein was immobilized onto a PVDF membrane using dot blot equipment (Cleaver Scientific, Rugby, UK). This was followed by a blocking step and incubation with the primary antibody. The primary antibodies were anti-FOXO3 (NBP2-16521), anti-FOXO3 (pSer253) (NBP2-67521), anti-NRF2 (NBP1-32822), anti-NRF2 (pSer40) (NBP2-67465) (Novus Biologicals, Bio-Techne, Minneapolis, MN, USA), NOS3 (sc-654), SOD1 (sc-101523), SOD2 (sc133134) (Santa Cruz Biotechnology, Dallas, TX, USA), NOS3 (Ser1177) (#9571) (Cell Signaling Technology, Danvers, MA, USA), anti-Caspase 3 (ab13847; Abcam, Cambridge, UK), anti-Caspase 8 (ab108333; Abcam, Cambridge, UK), anti-BID (ab62469; Abcam, Cambridge, UK), t-BID (ab10640; Abcam, Cambridge, UK), and *E. coli* protein (1/1000; ab25823; Abcam, Cambridge, UK).

The same secondary antibodies used in the Western blot experiments were also used here. For quantification, three independent biological replicates (*n* = 3 distinct liver samples) were analyzed per experimental condition.

Both Western blots and antibody arrays were visualized using enhanced chemiluminescence. Signal detection was carried out with an Omega Lum G imaging system (Aplegen, Gel Company, San Francisco, CA, USA), which is equipped with a CCD camera. Quantification of the signals was then performed using Licor Image Studio Lite version 5.2. Three Western blots were analyzed for each condition.

### 2.7. Statistical Analysis

The gut microbiota of the mice in all groups was analyzed using high-throughput sequencing (average number of reads ± SEM = 152,411 ± 47,828). Microbial diversity analyses were performed by clustering sequence tags into groups of defined sequence variation. *α*-Diversity measurements (observed OTUs, Chao 1, Shannon diversity index or SDI, and inverted Simpson index) and *β*-diversity measurements (Jaccard, Bray–Curtis, UniFrac, and weighted UniFrac) were analyzed using a blocked analysis of variance. The relative abundance of bacteria was compared with a MULTINOVA using the Jaccard and unweighted UniFrac similarity measures to construct distance metrics using QIIME 2 (Quantitative Insights Into Microbial Ecology 2) v 2019.7. All analyses were conducted using the R programming language in FROGS and GraphPad Prism 9 where ordinary one-way Anova and Student’s t-test were employed to determine statistical significance among experimental groups. A *p* value less than 0.05 was considered statistically significant.

## 3. Results

### 3.1. Gut Microbiota

**Figure 1:**
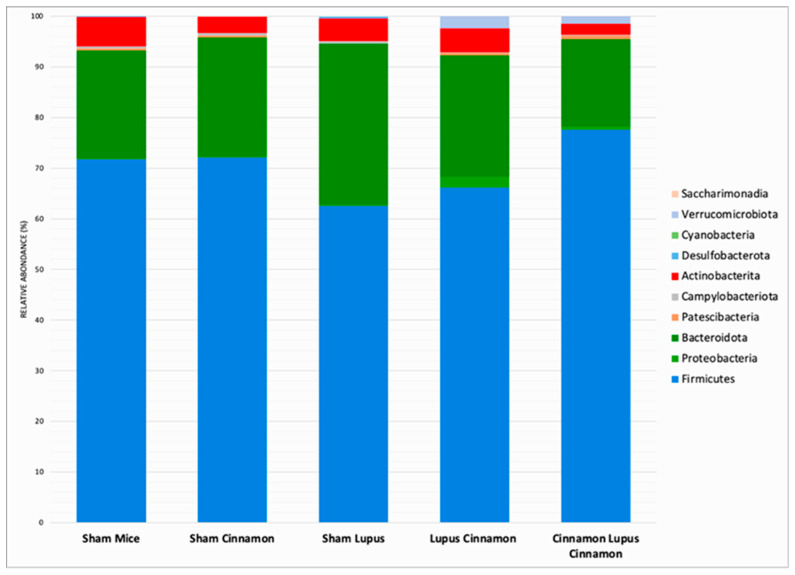
Gut microbiota colonization comparison at the phylum level of normal mice (Sham Mice), mice treated with cinnamon (Sham Cinnamon), mice with induced lupus (Sham Lupus), mice with induced lupus treated with cinnamon (Lupus Cinnamon), and mice supplemented with cinnamon prior to inducing lupus, then treated with cinnamon (Cinnamon Lupus Cinnamon).

**Figure 2:**
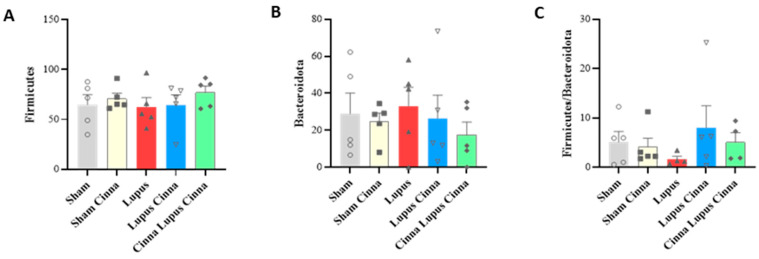
Firmicutes (**A**) and Bacteroidota (**B**) abundance with Firmicutes/Bacteroidota ratio level (**C**) of normal mice (Sham Mice), mice treated with cinnamon (Sham Cinna), mice with induced lupus (Sham Lupus), mice with induced lupus treated with cinnamon (Lupus Cinna), and mice supplemented with cinnamon prior to inducing lupus, then treated with cinnamon (Cinna Lupus Cinna). No statistical difference was observed between groups (*p* > 0.05).

The mice gut microbiota was dominated by three phyla: Firmicutes, Bacteroidota, and Actinobacterita (Figure 1). The Firmicutes/Bacteroidota ratio showed a downward trend in lupus mice compared to controls; in the LC and CLC groups, the mean ratio was higher than in the lupus group, though these differences did not reach statistical significance (*p* > 0.05) (Figure 2).

**Figure 3:**
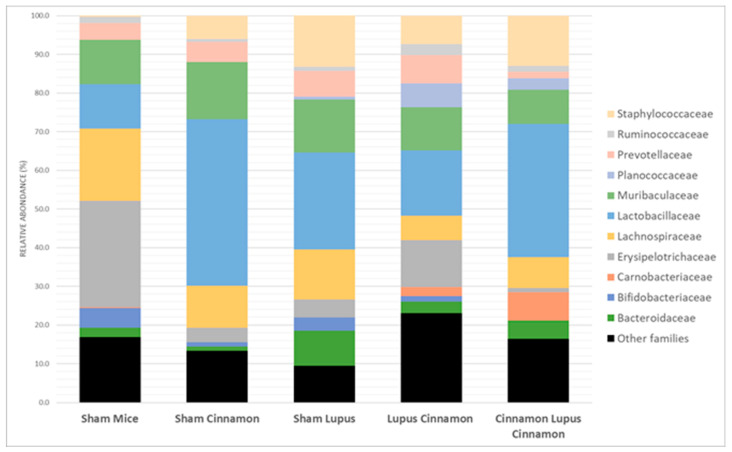
Gut microbiota colonization comparison at the family level of normal mice (Sham Mice), mice treated with cinnamon (Sham Cinnamon), mice with induced lupus (Sham Lupus), mice with induced lupus treated with cinnamon (Lupus Cinnamon), and mice supplemented with cinnamon prior to inducing lupus, then treated with cinnamon (Cinnamon Lupus Cinnamon).

**Figure 4:**
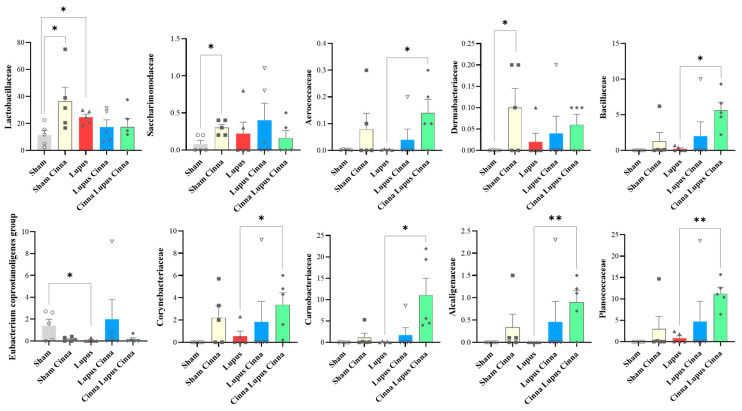
Relative abundance at the family level in the different groups: normal mice (Sham Mice), mice treated with cinnamon (Sham Cinna), mice with induced lupus (Sham Lupus), mice with induced lupus treated with cinnamon (Lupus Cinna), and mice supplemented with cinnamon prior to inducing lupus, then treated with cinnamon (Cinna Lupus Cinna). Only statistically significant differences are shown. *: *p* < 0.05. **: *p* < 0.01.

At the family level, the gut microbiota was mainly colonized by Lactobacillaceae, Lachnospiraceae, Erysipelotrichaceae, Muribaculaceae, Bacteroidaceae, Prevotellaceae, and Bifidobacteriaceae (Figure 3). Erysipelotrichaceae seems to be more represented in the control group (S) than in the lupus group. Although not statistically significant, cinnamon supplementation seems to decrease Lachnospiraceae. In addition, the obtained results showed Lactobacillaceae was significantly lower in lupus mice in comparison with the control group, while significantly increasing in the control group supplemented with cinnamon (Figure 4). Eubacterium coprostanoligenes was significantly decreased in the lupus group. Saccharimonodaceae was significantly increased by cinnamon in the control groups. Carnobacteriaceae, Planococcaceae, Aerococcaceae, Bacillaceae, Flavobacteriaceae, Corynebacteriaceae, Alcaligenaceae, Aeromonaceae, and Micrococcaceae were significantly increased in the CLC group in comparison to the lupus group.

**Figure 5:**
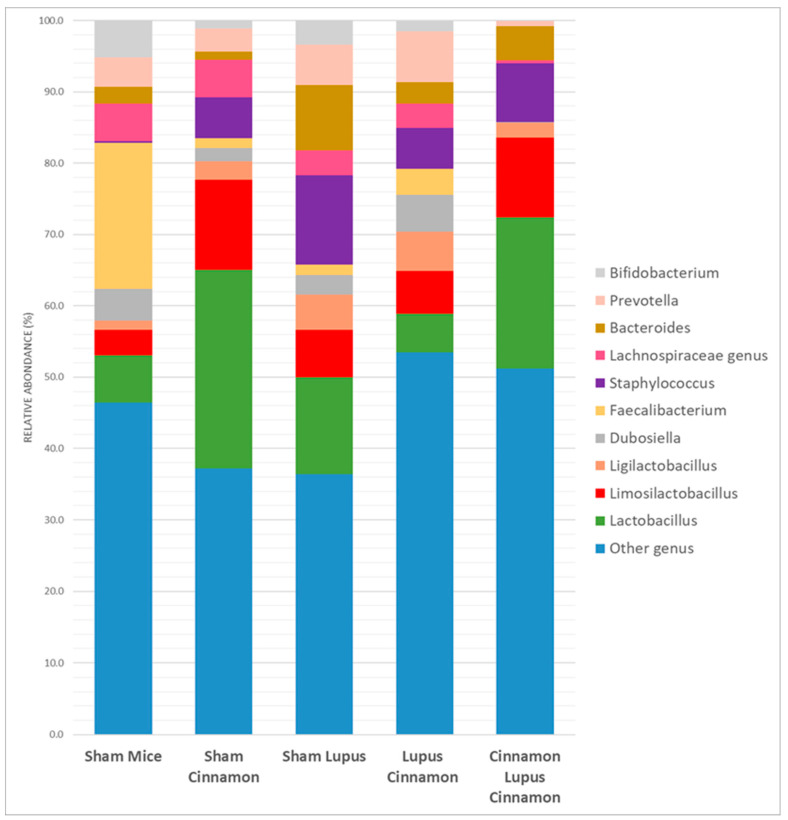
Gut microbiota colonization comparison at the genus level of normal mice (Sham Mice), mice treated with cinnamon (Sham Cinnamon), mice with induced lupus (Sham Lupus), mice with induced lupus treated with cinnamon (Lupus Cinnamon), and mice supplemented with cinnamon prior to inducing lupus, then treated with cinnamon (Cinnamon Lupus Cinnamon).

At the genus level, sham mice supplemented with cinnamon were more colonized by *Lactobacillus* (27.9%) compared to non-supplemented mice (6.7%) and mice with induced lupus (13.6%) (Figure 5 and Table 1). Preventive treatment (Cinna Lupus Cinna 21.2%) maintained *Lactobacillus* levels (21.2%) comparable to the sham cinnamon group, whereas the therapeutic regimen (Lupus Cinna) resulted in a lower abundance (5.4%) that was statistically different from the untreated lupus group (Figure 5 and Table 1). The genus *Limosilactobacillus* followed a very similar pattern to Lactobacillus (Figure 5 and Table 1).

**Figure 6:**
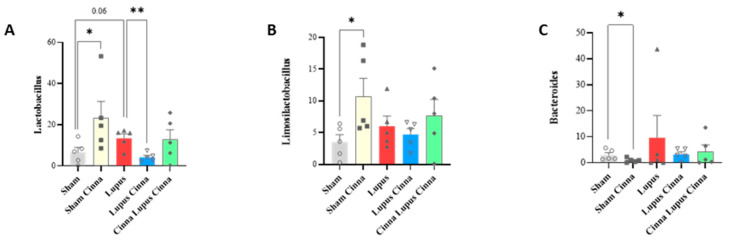
Relative abundance at the genus level (**A**): *Lactobacillus*; (**B**): *Limosilactobacillus*; (**C**): *Bacteroides*) in the different groups: normal mice (Sham Mice), mice treated with cinnamon (Sham Cinna), mice with induced lupus (Sham Lupus), mice with induced lupus treated with cinnamon (Lupus Cinna), and mice supplemented with cinnamon prior to inducing lupus, then treated with cinnamon (Cinna Lupus Cinna). Only statistically significant differences are shown. *: *p* < 0.05. **: *p* < 0.01.

Furthermore, *Faecalibacterium* was the highest in sham mice when compared to other groups (Table 1). On the other hand, mice with induced lupus were more colonized by *Staphylococcus* and *Bacteroides* than mice without lupus or with induced lupus supplemented with cinnamon. In addition, *Bifidobacterium* was mainly detected in sham mice regardless of lupus induction and cinnamon supplementation (Figure 6).

### 3.2. Effect of Lupus and Cinnamon on Microbial Diversity

Samples collected from the five groups were analyzed and compared in terms of α-diversity metrics (observed OTU richness, Chao-1, Shannon diversity index, and inverted Simpson). All the metrics did not differ significantly between sham mice, and mice supplemented with cinnamon with or without induced lupus (Supplementary Material Figure S1).

These results show that the number of species did not variate significantly in supplemented mice or mice with induced lupus. However, cinnamon intake increased slightly bacterial richness and diversity when compared to sham mice.

### 3.3. Effect of Lupus and Cinnamon on Microbial Communities

Following binning of the sequences into operational taxonomic units (OTUs) based on 97% sequence identity, comparisons were made using principal coordinates analysis (PCoA) based on weighted UniFrac distances (Supplementary Material Figure S2). Each sample corresponding to microbial communities from sham mice with or without cinnamon supplementation and lupus induction clustered tightly and separated on the second principal axis (P2). Samples clustered tightly but did not separate on the first principal axis (P1).

Continued analysis of the microbial communities from these mice did not show the observed divergence of the lupus group when compared to controls, particularly on the first principal axis (P1). Unweighted UniFrac analysis and Jaccard analysis did not reveal any difference in β-diversity by inducing lupus and by supplementing with cinnamon, showing interindividual diversity (Supplementary Material Figure S2).

### 3.4. KEGG Bioinformatic Analysis

**Figure 7:**
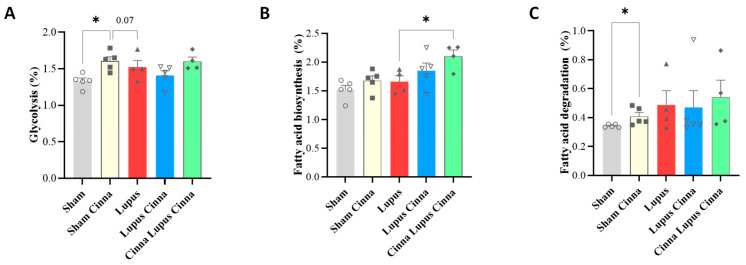
Relative percentage of the pathway’s distribution KEGG (**A**): glycolysis; (**B**): fatty acid biosynthesis; (**C**): fatty acid degradation in the different groups: normal mice (Sham Mice), mice treated with cinnamon (Sham Cinna), mice with induced lupus (Sham Lupus), mice with induced lupus treated with cinnamon (Lupus Cinna), and mice supplemented with cinnamon prior to inducing lupus, then treated with cinnamon (Cinna Lupus Cinna). Only statistically significant differences are shown. *: *p* < 0.05.

Cinnamon significantly enhances the glycolytic pathway in comparison to sham and lupus mice (Supplementary Figure S3). It also increases the fatty acid degradation pathway in comparison to the sham group. Lupus mice supplemented by cinnamon for a longer period (Cinna Lupus Cinna) exhibit significantly increased fatty acid biosynthesis versus lupus mice (Figure 7).

### 3.5. Escherichia coli Protein Expression in Liver Tissue

**Figure 8:**
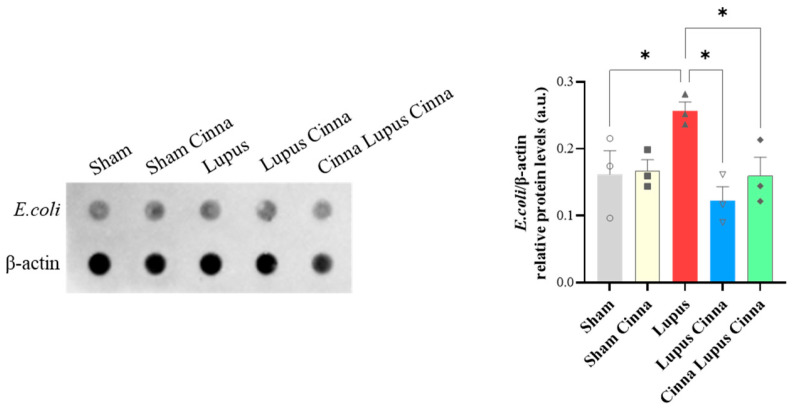
Antibody array and quantification of *E. coli* protein with β-actin in liver tissue by microarray measurement in different groups. *: *p* < 0.05.

Microarray studies reveal significantly increased *Escherichia coli* (*E. coli*) relative protein levels in lupus mice livers when comparing to sham mice; this increase was significantly reversed in cinnamon supplemented lupus groups (Figure 8).

### 3.6. TLR-4, TLR-7, and NFκB in the Liver

**Figure 9:**
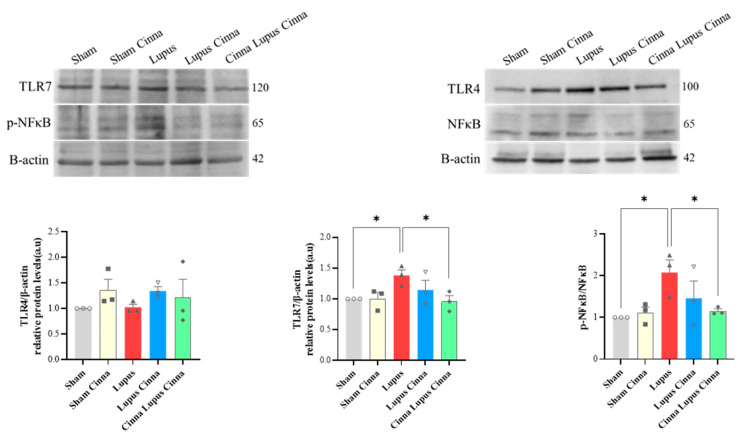
Western blot and quantification of TLR4, TLR7, and p-NFκB / NFκB with β-actin in liver tissue of different groups. *: *p* < 0.05.

Protein expression of TLR-7 and p-NFκB/NFκB in the liver tissue of lupus mice was significantly higher than that of the sham group. Preventive cinnamon supplementation (Cinna Lupus Cinna) significantly restored the expression of TLR-7 and p-NFκB/ NFκB. Protein expression of TLR4 was not significantly different between groups (Figure 9).

### 3.7. Oxidative Stress and Apoptosis Markers in the Liver

**Figure 10:**
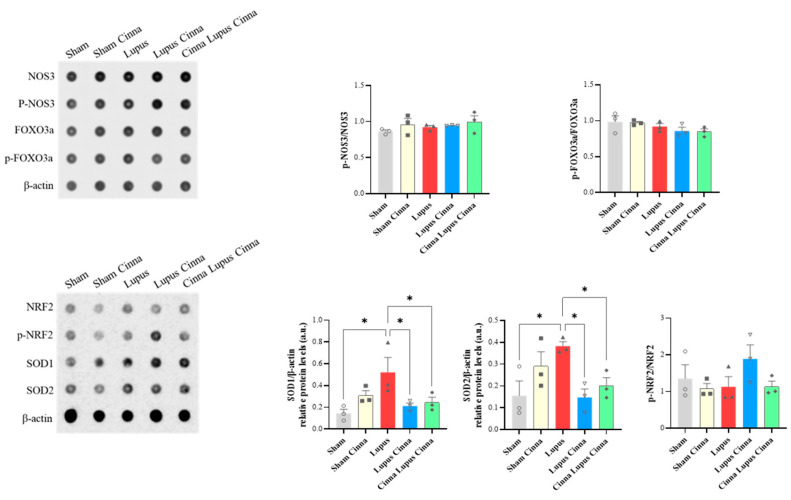
Antibody array quantification of p-NOS3/p-NOS3, p-FOXO3a/p-FOXO3a, p-NRF2/NRF2, SOD1, and SOD2 with βactin in liver tissue of the different groups. *: *p* < 0.05.

SOD1/β-actin and SOD2/β-actin were significantly increased in the liver tissue of lupus mice. Supplementation by cinnamon (LC and CLC) significantly reduced this increase (Figure 10). Expression of p-NOS3/p-NOS3, p-FOXO3a/p-FOXO3a, and p-NRF2/NRF2 did not differ significantly between groups. Furthermore, no significant change was found in apoptosis markers (Caspase 3, Caspase 8, t-BID/BID expression).

## 4. Discussion

The obtained results show, for the first time, that the cinnamon supplementation modulates intestinal microbiota, prevents *E. coli* liver translocation, calms down inflammatory pathways activation in liver tissue, and exhibits an ameliorative effect on hepatic oxidative stress in imiquimod-induced lupus.

Imiquimod-induced lupus is a well-established and widely accepted model for inducing systemic autoimmunity that mirrors key features of human lupus. As demonstrated by Yokogawa et al. (2014), the topical application of IMQ in mice leads to the development of systemic manifestations, including splenomegaly, lymphadenopathy, circulating autoantibodies (anti-ssDNA and anti-Sm/RNP), and a systemic type I interferon signature [8].

Our study is an extension of our previous investigations of this model with documented renal and brain lupus-related lesions and dysfunction and a documented ameliorative effect of cinnamon [7,11].

The oral cinnamon dosing of 200 mg/kg was chosen as it is well-supported by prior research to be a non-toxic and therapeutically effective concentration for eliciting both metabolic and anti-inflammatory benefits [9,10]. This evidence-based approach provides a solid foundation for its use in our study. In addition, although the precise intake of cinnamon was not monitored by pharmacokinetic analysis of its active metabolites in plasma, the administration method aligns with common practices in nutritional studies and was chosen to minimize animal stress [17,18]. Of note is that every mouse was observed for the complete consumption of the daily dose. Future research could incorporate such analyses to better define the dose–response relationship.

Since no single experimental model of lupus encompasses all aspects of the disease, various models exhibit distinct disease phenotypes. Likewise, the role of the microbiota in lupus seems to vary depending on the specific mouse model studied.

Firmicutes and Bacteroidota, the two dominant bacterial phyla in the gastrointestinal tract, have gained significant attention in recent years in various diseases. The Firmicutes/Bacteroidota (F/B) ratio is recognized as a crucial factor in maintaining intestinal homeostasis. An altered F/B ratio is considered a marker of dysbiosis, with an increase commonly linked to obesity and a decrease associated with inflammatory bowel disease [19].

A lower Firmicutes/Bacteroidota (F/B) ratio in MRL/lpr and NZBWF1 mice is linked to early disease initiation [20,21,22]. In human patients, a lower F/B ratio is observed in lupus in comparison to healthy subjects [23,24]. In addition, active lupus patients show a lower F/B ratio [25,26]. In our experimental conditions, a tendency towards a decreased F/B ratio was observed in lupus mice but reversed by cinnamon supplementation, especially in the preventive group.

MRL/lpr mice have reduced Lactobacillaceae and increased Lachnospiraceae [6]. Lachnospiraceae is associated with more severe lupus symptoms in this spontaneous model [27]. In our study, Lactobacillaceae was significantly lower in lupus mice in comparison with the control group, while significantly increasing in the control group supplemented with cinnamon. Furthermore, cinnamon supplementation favors a shift toward decreased Lachnospiraceae in sham and lupus treated mice.

At the genus level, cinnamon supplementation seems to enhance Lactobacillus abundance; this is in line with the prebiotic potential of cinnamon bark on Lactobacillus [28]. Several Lactobacilli are able to utilize phenolic acids (by decarboxylation and/or reduction reactions) and tolerate their presence much more than other members of the GM like *Clostridium* and *Bacteroides* members [29]. The relationship between systemic lupus erythematosus and *Lactobacillus* is complex and strain-dependent. Reviews show that *Lactobacillus* genera can be either decreased or increased in SLE cohorts and animal models, and different *Lactobacillus* strains may have protective immunomodulatory effects or, in some contexts, exacerbate autoimmunity [30]. Our results suggest a clear timing effect: cinnamon given before lupus induction maintains higher *Lactobacillus* abundance (21.2%), which was decreased when cinnamon is given after lupus induction (5.4%). This fits a plausible mechanism seen in the literature: cinnamon can shape the baseline microbiota and mucosal barrier [31], promoting colonization resistance and supporting beneficial taxa when applied prophylactically [32], but its antimicrobial constituents or altered host environment post-disease induction may differ; while preventive cinnamon alters the gut environment (pH, mucus, metabolites), favoring *Lactobacillus* expansion prior to immune challenge, post-induction cinnamon probably interacts with an inflamed mucosa or altered community and exerts antimicrobial effects that disproportionately reduce *Lactobacillus* in that pathological context. While modulating *Lactobacillus* levels may have a disease modifying effect, the efficacy of cinnamon may be mediated also by alternative mechanisms. These likely include the direct downregulation of hepatic TLR/NFkB signaling and the modulation of other microbial taxa, rather than solely depending on *Lactobacillus* restoration.

*Limosilactobacillus* is a genus of lactic acid bacteria that is both thermophilic and heterofermentative; it was established in 2020 as an independent genus following its separation from the genus *Lactobacillus* [33]. Data on the effect of *Limosilactobacillus reuteri* in lupus is limited and may seem contradictory [34]. In NZB/WF1 mice, pre-treatment with *L. reuteri* exhibits an ameliorative effect on survival, cardiovascular lesions, and liver proinflammatory cytokines IL-1 β, IL-6, and TNF-α [35,36]. In an opposite way, *L. reuteri* exacerbates lupus-like disease in a TLR7-dependant model [37]; however, the pathogenic effect of L. reuteri in the latter study was observed under specific-pathogen-free or germ-free conditions, and thus is not comparable to the effect of L. reuteri in the real, complex intestinal environment. In our study, Limosilactobacillus was found to be more abundant in sham mice supplemented with cinnamon than in non-supplemented mice and mice with induced lupus. Identifying *Lactobacillus* and *Limosilactobacillus* at the species or strain level by shotgun metagenomics or species-specific qPCR would be useful to clarify these heterogeneous results across studies and to determine whether the expanded taxa are immunoregulatory strains (e.g., *L. rhamnosus*, *L. reuteri*).

On the other hand, Staphylococcus and Bacteroides were increased in the lupus group in our study similarly to other human and animal studies [38].

Our experimental model did not reveal changes in alpha and beta diversity attributed to lupus induction or cinnamon supplementation. While some authors documented significant diversity shifts in an imiquimod-induced model [20,39], others did not find differences in the ecological parameters in this same model [20]. Sample size, environmental variability, and other factors may contribute to this discrepancy.

Cinnamon’s effect on intestinal microbiota diversity is heterogeneous across studies depending on formulation, specific extraction, and the animal or clinical conditions: while cinnamon oil and cinnamaldehyde microcapsules modulate diversity and richness of intestinal microbiota in mice [40,41] and cinnamon water extract increases alpha diversity in humans with diarrhea [42], cinnamaldehyde has not shown an impact on alpha and beta diversity in early weaned rats [32], and consumption of dietary polyphenols (including cinnamon compounds) in humans does not modify diversity measures [43]. In our study, cinnamon supplementation did not result in diversity modification. This suggests that cinnamon’s prebiotic effects might be more targeted towards specific microbial taxa rather than causing a broad modification in the richness of the gut microbiota. Future studies with a larger sample size may be needed to verify these findings.

Cinnamon’s effect on gut microbiota was reflected also by metabolic pathways modification in KEGG bioinformatic analysis. Increased glycolytic pathway may indicate positive impact of cinnamon on bacterial populations relying heavily on glycolysis, such as *Lactobacillus* [44]. The effect on fatty acid degradation may be related to cinnamon’s favoring of short-chain fatty acid-producing bacteria [45]. Increasing fatty acid biosynthesis in lupus mice supplemented by cinnamon for a long period is more intriguing and necessitates further investigation in future studies.

These intestinal microbiota modifications related to lupus and cinnamon supplementation were accompanied by alteration in gut–liver axis key components. We previously demonstrated gut permeability dysfunction with liver *E. coli* translocation and increased TLR4, TLR7, and pNFκB/NFκB liver expression in imiquimod-induced lupus [2]. Our study’s primary objective was not to demonstrate that bacterial translocation causes or exacerbates lupus; this link has already been compellingly established in landmark studies that identified *Enterococcus gallinarum* translocation as a trigger for autoimmunity in genetically predisposed mice [46]. Instead, our goal was to leverage the validated model of systemic inflammation induced by imiquimod to investigate the effects of cinnamon on the gut–liver axis. Moreover, our previous work showed that intestinal tight junctions’ expression is decreased in this model and rescued by cinnamon supplementation [37]. Our present study confirms the presence of a gut–liver axis in this lupus model and adds new data on the protective effect of cinnamon on gut permeability and liver activation of TLR7 and NFκB. This consolidates the beneficial effect of cinnamon on gut barrier function observed in other metabolic or inflammatory conditions [32]. The reduction in bacterial translocation cannot be solely attributed to a direct effect of specific taxa modification (like *Lactobacillus*) by cinnamon. Instead, it is likely the result of a multifaceted mechanism involving both global shifts in the microbiota and the direct reinforcement of the gut epithelial barrier by cinnamon.

Very few studies investigated liver-specific oxidative stress in lupus. Mitochondrial dysfunction was described in lupus-prone mice as an early event [47]. Our microarray screening of key antioxidant markers in the liver revealed increased SOD1 and SOD2 expression in lupus mice. Upregulation of these enzymes may reflect compensatory mechanisms of coping with high oxidative stress [48,49]. Reversing this increased expression with cinnamon may be a surrogate marker of the antioxidant effect of cinnamon and its modulating effect on TLR7-mediated oxidative stress [50].

Our study has certain limitations. First, the detection of *E. coli* in the liver relied on antibody arrays targeting bacterial proteins; while this confirms the translocation of bacterial antigens indicative of barrier dysfunction, it does not confirm the presence of viable, replicating bacteria. Second, while sample sizes (*n* = 10) were sufficient to detect some taxonomic shifts, high inter-individual variability in microbiome data and the possible underpowerment of the study may have obscured more subtle changes, resulting in trends that did not reach statistical significance. Finally, discrepancies between our findings and other lupus models highlight the heterogeneity of lupus, suggesting that microbiota-targeted interventions may need to be tailored to specific disease endotypes.

## 5. Conclusions

In conclusion, *Cinnamomum cassia* modulates specific elements of the gut–liver axis in imiquimod-induced lupus. It effectively reduces the hepatic translocation of bacterial products and downregulates inflammatory and oxidative markers. While global microbiome diversity was not significantly altered, specific changes suggest a targeted prebiotic effect. These findings offer a perspective on nutritional interventions as adjunct therapies in lupus management.

## Figures and Tables

**Table 1:** Relative abondance of intestinal microbiota at the genus level.

	Sham Mice	Sham Cinnamon	Sham Lupus	Lupus Cinnamon	Cinnamon Lupus Cinnamon
Other genus	46.4	37.2	36.4	53.5	51.2
*Lactobacillus*	6.7	27.9	13.6	5.4	21.2
*Limosilactobacillus*	3.5	12.6	6.6	6.0	11.2
*Ligilactobacillus*	1.3	2.5	4.9	5.5	2.0
*Dubosiella*	4.5	1.9	2.8	5.2	0.1
*Faecalibacterium*	20.5	1.4	1.5	3.6	0.0
*Staphylococcus*	0.3	5.8	12.5	5.8	8.2
*Lachnospiraceae genus*	5.2	5.3	3.5	3.4	0.4
*Bacteroides*	2.4	1.1	9.1	3.0	4.7
*Prevotella*	4.1	3.2	5.7	7.1	0.8
*Bifidobacterium*	5.1	1.2	3.4	1.5	0.0

## Data Availability

The original contributions presented in this study are included in the article/Supplementary Material. Further inquiries can be directed to the corresponding author.

## Supplementary Materials

The following supporting information can be downloaded at: https://www.mdpi.com/article/10.3390/biomedicines14010006/s1, Figure S1: α-Diversity (observed OTU richness, Chao-1, Shannon diversity index, and inverted Simpson index) of normal mice (Sham Mice), mice treated with cinnamon (Sham Cinnamon), mice with induced lupus (Sham Lupus), mice with induced lupus treated with cinnamon (Lupus Cinnamon), and mice supplemented with cinnamon prior to inducing lupus, then treated with cinnamon (Cinnamon Lupus Cinnamon); Figure S2: 16S rRNA-based analysis by weighted UniFrac normal mice (Sham Mice), mice treated with cinnamon (Sham Cinnamon), mice with induced lupus (Sham Lupus), mice with induced lupus treated with cinnamon (Lupus Cinnamon), and mice supplemented with cinnamon prior to inducing lupus, then treated with cinnamon (Cinnamon Lupus Cinnamon). Figure S3: Pathways distribution KEGG in the different groups.
