# Supplementary material for: Cinnamomum cassia Modulates Key Players of Gut–Liver Axis in Murine Lupus
Source: Biomedicines. 2025 Dec 19;14(1):6. doi: 10.3390/biomedicines14010006 (PMC12837613; doi:10.3390/biomedicines14010006)
Supplement: Supplementary file 1 [file biomedicines-14-00006-s001.zip › biomedicines-3997645-supplementary.pdf]

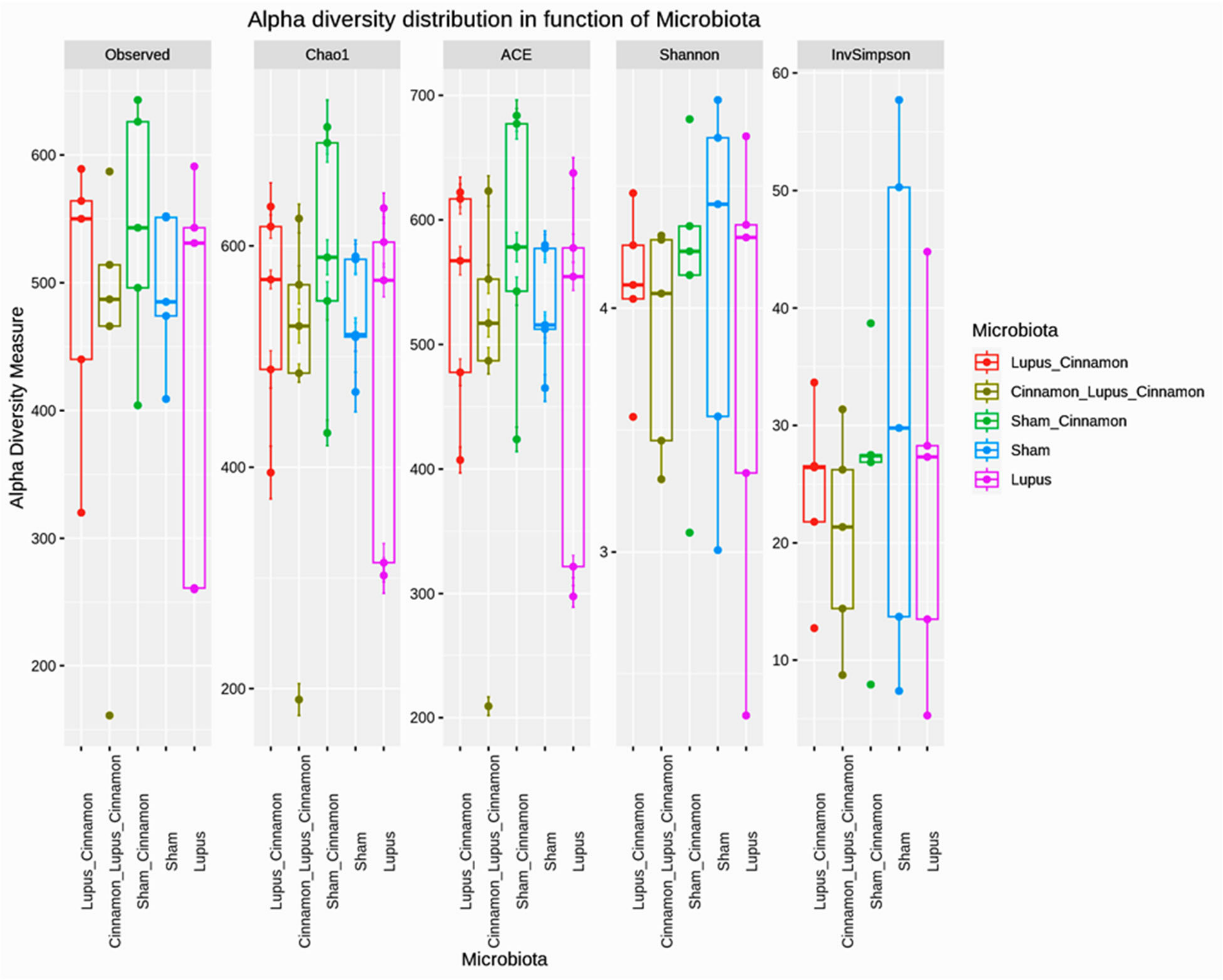

Supplementary Figure S1. α-Diversity (observed OTU richness, Chao-1, Shannon diversity index, and inverted Simpson index) of normal mice (Sham Mice), mice treated with cinnamon (Sham Cinnamon), mice with induced lupus (Sham Lupus), mice with induced lupus treated with cinnamon (Lupus Cinnamon), and mice supplemented with cinnamon prior to inducing lupus, then treated with cinnamon (Cinnamon Lupus Cinnamon).

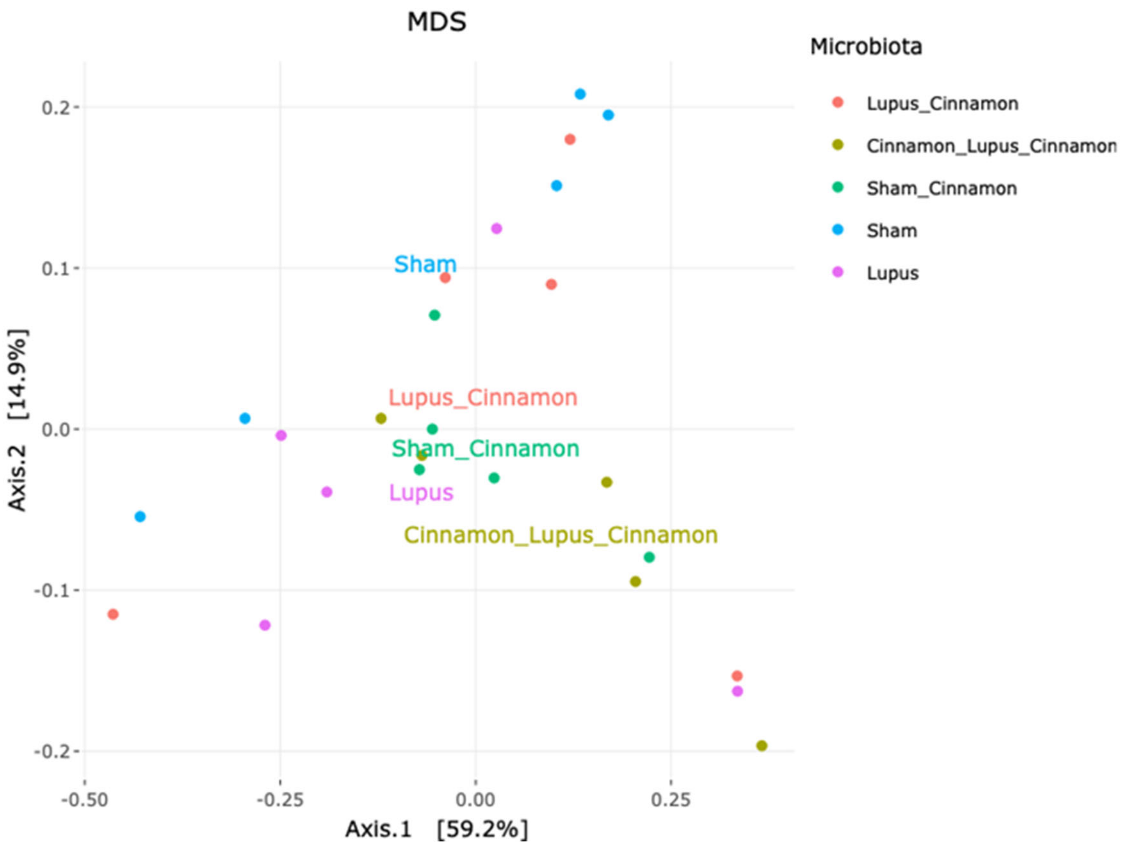

Supplementary Figure S2. 16S rRNA-based analysis by weighted UniFrac normal mice (Sham Mice), mice treated with cinnamon (Sham Cinnamon), mice with induced lupus (Sham Lupus), mice with induced lupus treated with cinnamon (Lupus Cinnamon), and mice supplemented with cinnamon prior to inducing lupus, then treated with cinnamon (Cinnamon Lupus Cinnamon).

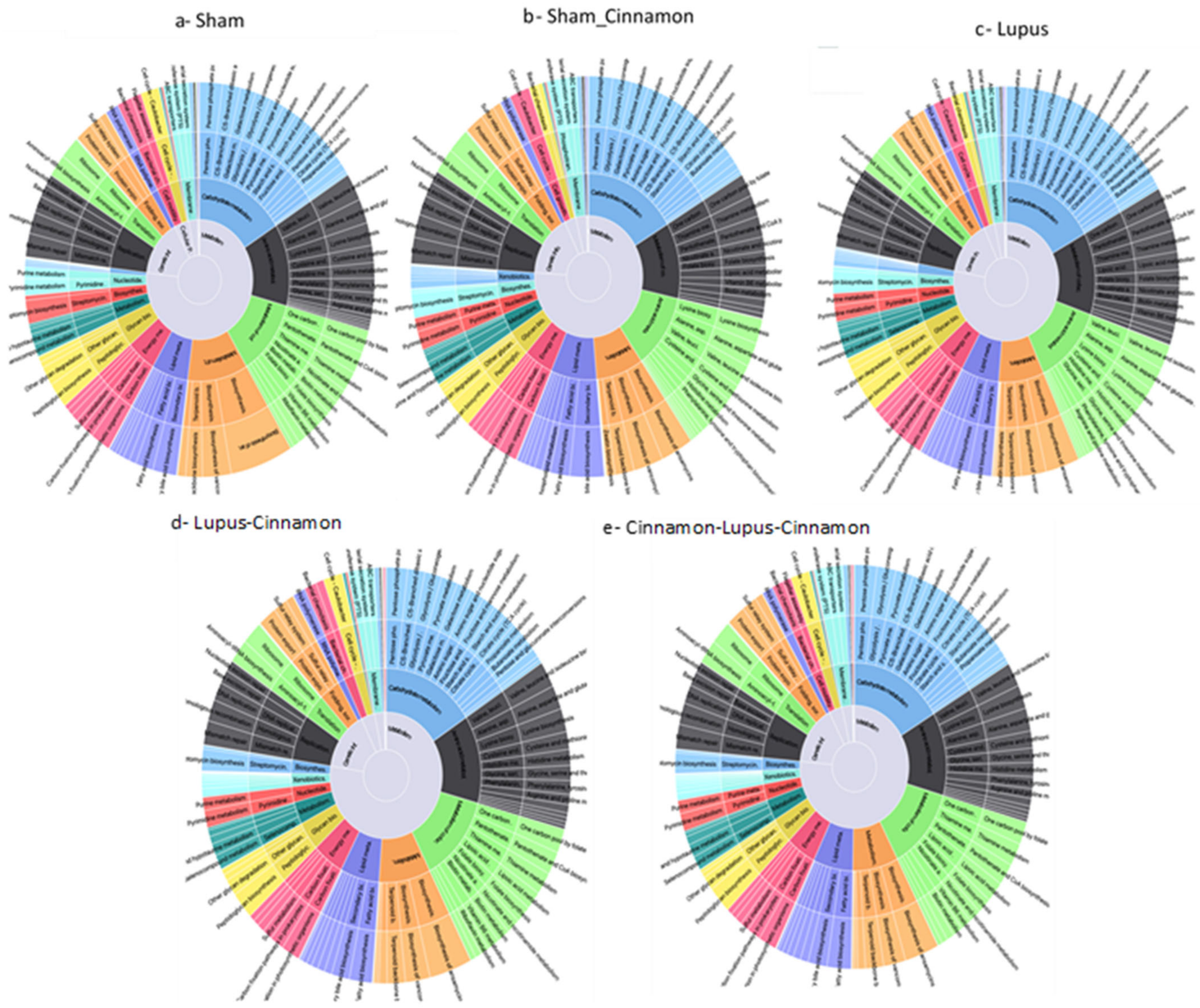

Supplementary Figure S3. Pathways distribution KEGG in the different groups.
